# Hidden reservoirs of infection: prevalence and risk factors of asymptomatic malaria in a high-endemic region of Zambia

**DOI:** 10.1186/s12936-025-05472-w

**Published:** 2025-07-08

**Authors:** Wisdom Silwamba, David Chisompola, John Nzobokela, Martin Chakulya, Lombe Kabwe, Kingsley Tembo

**Affiliations:** 1 Pathology Laboratory Department, Mwandi Mission Hospital, Mwandi, Zambia; 2 Pathology Laboratory Department, Arthur Davison Children’s Hospital, Ndola, Zambia; 3 Pathology Laboratory Department, Ndola Teaching Hospital, Ndola, Zambia; 4 Healit Research International and Trace Research & Innovation, Untold Global Healit Zambia, Lusaka, Zambia; 5 Pathology and Microbiology Department, Mulungushi University School of Medicine and Health Sciences, Livingstone, Zambia

**Keywords:** Asymptomatic Malaria, Prevalence, Risk factors, Endemic, Mwandi District, Public Health, Zambia

## Abstract

**Background:**

Malaria remains a significant global health challenge, particularly in sub-Saharan Africa (SSA), where asymptomatic cases contribute to ongoing transmission and hinder elimination efforts. Asymptomatic individuals act as hidden reservoirs, sustaining onward malaria transmission. This study aimed to determine the prevalence and risk factors of asymptomatic malaria in Mwandi District, Zambia.

**Methods:**

A cross-sectional study was conducted between January to May 2024 in Mwandi District. Blood samples were collected for malaria diagnosis and simultaneously tested using rapid diagnostic tests and Giemsa-stained blood smear microscopy techniques to detect *Plasmodium* infections. Structured questionnaires were administered to gather demographic data and information on potential risk factors. Descriptive statistics were used to summarize the data while categorical variables were compared using the chi-square test or Fisher’s exact test. Logistic regression was used to assess associations between outcomes and independent variables, with statistical significance set at *p* < 0.05.

**Results:**

A total of 370 participants were enrolled in the study, with females comprising the majority (52.4%) and a median age of 24 years (IQR: 9–30). The overall prevalence of asymptomatic malaria was (4.1%), as determined by microscopy. Logistic regression analysis showed that females had significantly lower odds of asymptomatic malaria compared to males (AOR: 0.20, 95% CI 0.05–0.68; *p* = 0.010). Additionally, participants residing in Matoya and Sikute were more likely to have asymptomatic malaria, with adjusted odds ratios of 4.56 (95% CI 1.10–18.80; *p* = 0.036) and 4.72 (95% CI 1.03–21.50; *p* = 0.045), respectively. No significant associations were found with insecticide-treated net use, indoor residual spraying, or socioeconomic status.

**Conclusion:**

The findings highlight the need for targeted surveillance and interventions in high-risk groups and locations to curb silent transmission. Despite limitations in diagnostic sensitivity, the study underscores the importance of integrating asymptomatic malaria screening into control programmes to advance elimination efforts in Zambia and similar endemic regions.

## Background

Malaria, a life-threatening disease transmitted by infected female *Anopheles* mosquitoes, remains a significant global health concern. Sub-Saharan Africa (SSA) bears the brunt of this burden, accounting for the majority of severe cases and deaths associated with the disease [1]. While symptomatic malaria is diagnosed and treated, asymptomatic cases often go undetected, posing a substantial challenge to elimination efforts [2]. These asymptomatic carriers, who harbour *Plasmodium* parasites without exhibiting clinical symptoms, play a crucial role in the silent transmission of malaria, contributing to its continued spread in endemic regions [3]. Zambia, like many malaria-endemic countries, faces a high disease burden. Recent estimates indicate approximately 20,000 malaria cases occur daily in the country, with an average of four malaria-related deaths each day. A significant proportion of these cases are asymptomatic, particularly in regions where repeated exposure to infective mosquito bites has led to acquired immunity among the population [4, 5].

Asymptomatic malaria prevalence across SSA varies widely, with reported rates ranging from 5.03 to 34.7%, reflecting significant regional differences in transmission dynamics [2, 6–8]. More recently, a 2024 study from Côte d’Ivoire reported the highest prevalence of asymptomatic infections comprising 96.4% of all malaria infections [9]. This study emphasized the high prevalence of asymptomatic infections, particularly in school-age children, and highlighted the importance of sensitive diagnostic methods like polymerase chain reaction (PCR) for detecting sub-patent infections [9]. These findings highlight the widespread nature of asymptomatic malaria and underscore its implications for malaria control and elimination strategies.

*Plasmodium falciparum*, the most prevalent malaria parasite in SSA, is responsible for severe complications such as anaemia, cerebral malaria, organ failure, and death. Pregnant women and children under five years of age are particularly vulnerable to severe outcomes due to their weaker immune systems [4, 7]. Despite its critical role in sustaining transmission, asymptomatic malaria has received less attention compared to severe symptomatic and uncomplicated malaria. This lack of focus is partly due to the challenges associated with detecting asymptomatic infections, which require active surveillance and molecular diagnostic techniques to identify asymptomatic cases [7, 10–12].

In Zambia, particularly in high-transmission areas like Mwandi District, data on the prevalence and risk factors of asymptomatic malaria are limited. This knowledge gap poses a challenge to malaria control and elimination efforts, as asymptomatic carriers act as hidden reservoirs, contributing to ongoing transmission [13, 14]. Understanding the dynamics of asymptomatic malaria is crucial for developing effective strategies to reduce transmission and ultimately achieve malaria elimination.

Mwandi District, experiences persistent transmission due to favorable climatic conditions, including warm temperatures and dense vegetation, which provide ideal breeding grounds for *Anopheles* mosquitoes [15]. The district's geography, characterized by numerous water bodies and agricultural activities, further exacerbates the breeding of mosquitoes, thereby increasing the risk of malaria transmission [15, 16]. Malaria management in Zambia primarily focuses on treating symptomatic cases, with an emphasis on vector control strategies such as Indoor Residual Spraying (IRS) and the distribution of Insecticide-Treated Nets (ITNs) [17]. However, asymptomatic infections continue to pose a significant challenge, hindering efforts to achieve malaria elimination. This highlights the need for a deeper understanding of the prevalence and risk factors associated with asymptomatic malaria to inform targeted intervention strategies [18].

The importance of addressing asymptomatic malaria cannot be overstated. Asymptomatic carriers not only contribute to the ongoing transmission of malaria but also complicate efforts to monitor disease trends and evaluate the effectiveness of control measures. In regions with high levels of asymptomatic infections, the true burden of malaria may be underestimated, leading to inadequate resource allocation and ineffective intervention strategies [8, 19]. Therefore, it is essential to integrate asymptomatic malaria surveillance into existing malaria control programmes to ensure comprehensive coverage and accurate assessment of disease prevalence.

This study aimed to determine the prevalence of asymptomatic malaria and identify associated demographic, environmental, and behavioural risk factors in Matoya, Mwandi District, Zambia. By shedding light on this understudied aspect of malaria transmission, the findings will contribute to the development of more effective control and elimination strategies in high-burden regions. The study's outcomes will provide valuable insights into the epidemiology of asymptomatic malaria, helping policymakers and public health officials tailor interventions to address the specific needs of communities at risk. Ultimately, this research seeks to bridge the knowledge gap in asymptomatic malaria and support efforts to reduce malaria transmission, thereby moving closer to the goal of malaria elimination in Zambia and beyond.

## Methods

### Study design and study site

**Fig. 1:**
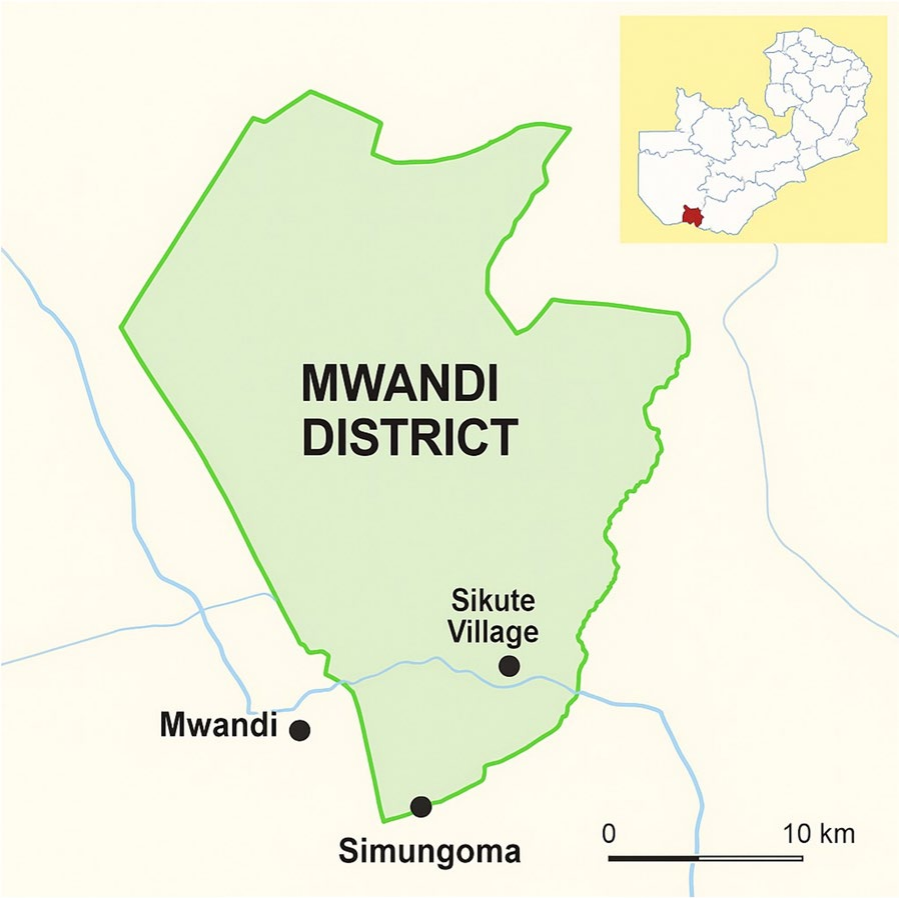
Geographical location of Mwandi District, Zambia

A cross-sectional study was conducted from January 2024 to May 2024 to assess the prevalence of asymptomatic malaria and identify associated factors in Mwandi District, Western Province, Zambia. Mwandi District is a malaria-endemic region located along the Zambezi River, approximately 140 km west of Livingstone and 65 km east of Sesheke [15]. It lies between longitudes 24.1° and 25.2° East of Greenwich and latitudes 16.6° and 17.8° South of the Equator [15] (Fig. 1). The district borders Namibia and is characterized by favorable ecological conditions, including warm temperatures, and dense vegetation, which contribute to persistent malaria transmission. The local population primarily engages in cattle keeping, farming, and fishing as their main economic activities. Matoya Rural Health Centre serves a catchment population of over 5,000 people, making it a suitable site for investigating asymptomatic malaria in a high-transmission setting.

### Inclusion and exclusion criteria

In this study, asymptomatic malaria was defined as the presence of *Plasmodium* parasites in peripheral blood, as detected by rapid diagnostic tests (RDTs) or microscopy, in individuals who did not exhibit any clinical symptoms of malaria at the time of recruitment [20]. Participants with symptomatic malaria, defined as the presence of fever (≥ 37.5 °C) or recent history of fever in the past 48 h, along with other clinical symptoms such as chills, headache, body aches, and general malaise [20, 21], were excluded from the study. Such cases were promptly referred to the nearest health facility for appropriate medical care. Individuals aged five years and older without clinical symptoms of malaria were eligible for inclusion. For participants aged 5–14 years, assent was obtained from their guardians along with informed consent, while those aged 15 years and older provided informed consent directly. Participants were also excluded if informed consent (for those aged 15 and above) or guardian assent (for those aged 5–14) was not granted. This screening process ensured that only truly asymptomatic individuals were enrolled, allowing for accurate assessment of asymptomatic malaria prevalence and associated factors in the community.

### Sample size

The required sample size (N) for this study was determined using the Cochran formula for cross-sectional studies [22]:

$$N=\frac{p(1-p)({Z}_{c}{)}^{2}}{{e}^{2}}$$

where:

Z = 1.96 (z-score for a 95% confidence level)

p = 0.50 (assumed prevalence from a prior study for maximum variability)

e = 0.05 (margin of error, 5%)

Substituting the values:

$$N=\frac{0.5(1-0.5)(1.96{)}^{2}}{{0.05}^{2}} =384.16=384$$

A minimum of **384** participants was required. However, due to logistical constraints and participant availability, the final sample size was 370, which is still statistically robust for the study.

### Sampling technique

A simple random sampling method with replacement was used to ensure an unbiased selection of participants [8]. The study population was first divided into smaller subpopulations, or clusters, based on geographic location (Matoya, Sikute, and Simungoma) to account for potential variations in malaria prevalence. Within each cluster, a random draw method (fishbowl technique) was employed to select a predetermined number of households. The selection process involved randomly drawing household identifiers from a pool, with replacement, ensuring that each household had an equal chance of being selected. Once a household was chosen, all eligible members were considered for malaria testing, with final participant selection based on predefined study inclusion criteria. This approach minimized selection bias while ensuring a representative sample of the population, enhancing the generalizability of the study findings.

### Specimen collection and processing

Malaria diagnosis was performed using both RDTs and microscopic examination of blood films. The SD Bioline malaria Ag P.f./Pan 05FK60 (Standard Diagnostics Inc., Suwon City, South Korea) RDT was used to detect malaria infections, following the manufacturer’s instructions to ensure accuracy. A drop of capillary blood was collected from each participant and applied to the test device, followed by the addition of the buffer solution, and results were read after 15 min based on the presence of control and test lines. Tests with no control line were deemed invalid and immediately repeated. To preserve test performance, RDT kits were stored in a dry environment at a controlled temperature of 25–28 °C [23]. Additionally, finger-prick blood samples were used to prepare thin and thick blood smears for microscopic examination to confirm malaria infection, determine parasite density, and identify *Plasmodium* species. The smears were air-dried for 10 min; thin films were fixed with 99.9% methanol and stained with a freshly prepared 10% Giemsa solution. Slides were examined under a microscope at 100X magnification using oil immersion, with two trained laboratory technologists independently evaluating each slide [23]. Any discrepancies between their assessments were resolved by a third, blinded and experienced microscopist to ensure diagnostic accuracy and minimize bias.

### Data collection procedures

The dependent variable in this study was asymptomatic malaria infection, categorized as either positive or negative. Independent variables included demographic and socio-economic factors such as age, sex, residency, occupation, ownership and use of ITNs, knowledge of malaria prevention, IRS practices, and socio-economic status [23, 24]. Data collection was conducted using a structured questionnaire administered to participants to gather relevant information on the independent variables.

### Data analysis

The data was entered, coded, and analyzed using the Statistical Package for the Social Sciences (SPSS), version 25. Proportions were calculated to estimate the prevalence of asymptomatic malaria. The normality of continuous data was assessed using the Gaussian distribution. Descriptive statistics, including means/medians and frequency distributions, were used to summarize the data. The chi-square test was applied to compare categorical groups, and when its assumptions were not met, the Fisher exact test was used. Logistic regression analysis was performed to evaluate the relationship between asymptomatic infection detected by microscopy and various independent variables. A *p*-value of < 0.05 was considered statistically significant in both bivariate and multivariable analyses, and all significant associations were reported with a 95% confidence interval.

## Results

### Social-demographic characteristics

A total of 370 participants were enrolled in the study, with females comprising the majority at 194 (52.4%) and the median age of participants was 24 years (IQR: 9–30) (Table 1). Nearly half of the participants, 184 (49.7%) were from Matoya Central, while children under the age of 15 accounted for 46.5% of the study population. Fishermen made up 163 (44.1%) of the participants. Additionally, 316 (85%) of the participants came from low-income households (Table 1). A statistically significant association was observed between asymptomatic malaria and location, with Simungoma recording the highest prevalence at 7 (6.9%, *p* = 0.050). Children under 15 years showed a significantly higher prevalence of asymptomatic malaria 9 (5.2%) compared to those over 15 years 6 (3.0%; *p* = 0.009). A significant association was also found with male sex 11 (6.3%; *p* = 0.041) (Table 1). However, no significant associations were observed between asymptomatic malaria and other factors, including the use of ITNs, IRS, socioeconomic status, knowledge of prevention methods, or occupation, as all corresponding *p*-values exceeded 0.05 (Table 1).

**Table 1:** Characteristics of the study population

Variables	Median (IQR)/Frequency (%)	Asymptomatic *Plasmodium falciparum* infection by Microscopy		P value
		Yes	No	
Age	24 (9, 30)	24 (9,30)	13 (10, 30)	0.9041
Gender				
Male	176 (47.6)	11 (6.3)	165 (93.8)	**0.041**
Female	194 (52.4)	4 (2.1)	190 (97.9)	
Area of Residence				
Matoya	184 (49.7)	3 (1.6)	181 (98.4)	**0.043**
Sikute	85 (23.0)	5 (5.9)	80 (94)	
*Simungoma*	101 (27.3)	7 (6.9)	94 (93.1)	
Indoor Residual Spraying				
No	34 (9.2)	2(5.9)	32 (94.1)	0.571
Yes	336 (90.8)	13 (3.9)	323 (95.9)	
ITNs				
No	336 (90.8)	13 (3.9)	323 (96.1)	0.571
Yes	34 (9.2)	2 (5.9)	32 (94.1)	
Prevention Knowledge				
Low	52 (14.1)	1 (1.9)	51 (98.0)	0.685
Moderate	273 (74.2)	10 (3.8)	263 (96.2)	
High	43 (11.7)	3 (7.5)	40 (92.5)	
Occupation				
Farmer	35 (9.5)	1 (2.9)	34 (97.1)	0.562
Fisherman	163 (44.1)	5 (3.1)	158 (96.9)	
NIL	172 (46.4)	9 (5.2)	163 (94.8)	
Social-economic Status				
Low (< 2000 Kwacha)	316 (85.4)	15 (4.8)	301 (95.2)	0.255
High (> 2000 Kwacha)	54 (14.6)	0 (0)	54 (100)	
Age category				
15 and below	172 (46.5)	9 (5.2)	163 (94.8)	**0.009**
Above 15	198 (53.5)	6 (3.0)	192 (97.0)	

Bold values indicate statistically significant associations (P < 0.05) based on chi-square/Fisher’s exact tests

### Prevalence of asymptomatic malaria

**Fig. 2:**
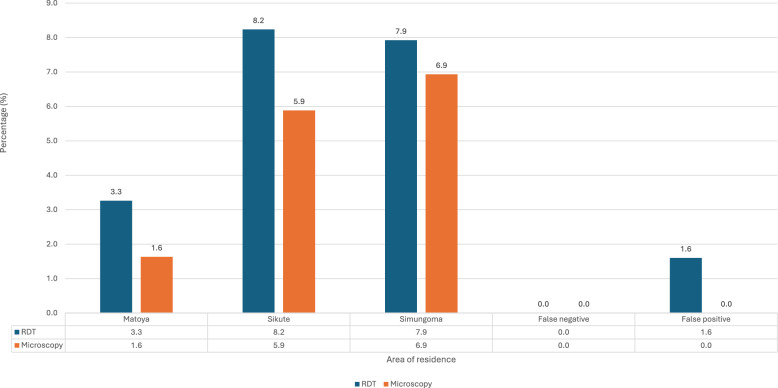
Prevalence of asymptomatic malaria by diagnostic method across study sites in Mwandi District

The overall prevalence of asymptomatic malaria based on microscopy was 15 (4.1%). Asymptomatic malaria infection varied by area of residence, Simungoma recorded the highest prevalence at 7 (6.9%), followed by Sikute at 5 (5.9%), and Matoya showing the lowest at 3 (1.6%) (Fig. 2). In contrast, the prevalence based on RDT was 5.6%, with Sikute showing the highest rate at 7 (8.2%), followed by Simungoma at 8 (7.9%), and Matoya again recording the lowest at 6 (3.3%) (Fig. 2). There were no cases of false negatives; however, 6 (1.6%) false positives were detected using RDT, with microscopy considered the gold standard.

### Factors associated with asymptomatic malaria by logistic regression

Variables such as area of residence, sex, and age category were selected for inclusion in both univariate and multivariate logistic regression analyses due to their statistical significance using microscopy results (*p* < 0.05). After adjusting for potential confounders, females had significantly lower odds of asymptomatic malaria compared to males (AOR: 0.20, 95% CI 0.05–0.68; *p* = 0.010) (Table 2). Participants from Matoya were significantly more likely to have asymptomatic malaria compared to those from other areas, with an adjusted odds ratio (AOR) of 4.56 (95% CI 1.10–18.80; *p* = 0.036). Similarly, participants from Sikute had increased odds of asymptomatic malaria, with an AOR of 4.72 (95% CI 1.03–21.5; *p* = 0.045). No statistically significant associations were observed between asymptomatic malaria and age, occupation, use of insecticide-treated nets (ITNs), indoor residual spraying (IRS), socioeconomic status, or malaria prevention knowledge (Table 2).

**Table 2:** Factors associated with asymptomatic malaria in logistic regression

Variable	OR (95% CI)	P value	AOR (95% CI)	P value
Sex				
Male	Ref		Ref	
Female	0.31 (0.09–1.01)	**0.052**	0.20 (0.05–0.68)	**0.010**
Area of residence				
Simungoma	Ref		Ref	
Sikute	3.77 (0.87–16.1)	0.074	4.72 (1.03–21.5)	**0.045**
Matoya	4.49 (1.13–17.7)	**0.032**	4.56 (1.10–18.8)	**0.036**
Age category				
15 and below	Ref		Ref	
Above 15	0.56 (0.19–1.62)	0.290	0.51 (0.16–1.58)	0.244

Bold values indicate statistically significant associations (P < 0.05) based on chi-square/Fisher’s exact tests or logistic regression

### Microscopy results for asymptomatic malaria-positive participants

Among the 15 participants who were positive by microscopy, the geometric mean parasite density was 765.7 parasites/µL (95% CI 490.9–1194.4) (Table 3). Among microscopy-positive cases, only one (0.27%) showed detectable gametocytes, with a gametocyte density of 14 gametocytes/µL (Table 3).

**Table 3:** Microscopy Results for Asymptomatic Malaria-Positive Participants

Variable	Value
Number of microscopy-positive cases	15
Geometric mean parasite density (parasites/µL)	765.7 (95% CI 490.9, 1194.4)
Number of participants with detectable gametocytes	1
Prevalence of detectable gametocytes	0.27%
Gametocyte density (gametocytes/µL)	14.0

^1^Only one participant had detectable gametocytes, which was insufficient to compute a geometric mean gametocyte density

## Discussion

Asymptomatic malaria poses a significant challenge to malaria control and elimination initiatives in the SSA. Since individuals with asymptomatic infections do not display noticeable symptoms, they rarely seek medical attention, inadvertently maintaining the cycle of malaria transmission by serving as a hidden reservoir for the parasite [18, 25]. This study provides critical insights into the prevalence and risk factors associated with asymptomatic malaria in Matoya, Mwandi District, Zambia.

The overall prevalence of asymptomatic malaria was (4.1%), this was consistent with 4.2% reported in Ethiopia [26]. However, it is lower than the 10.2% reported in north Ethiopia [27], 34.7% in Uganda, and 8% in Zambia's Southern Province over a decade ago [8, 28]. Recent studies have attributed the decline in the prevalence of asymptomatic malaria in high-burden regions in Zambia to the scaling up of ITN distribution and IRS campaigns [29]. Therefore, the lower prevalence observed in this study is consistent with recent trends in Zambia, where malaria control programs have intensified efforts to reduce transmission, and may in part reflect the impact of improved malaria control measures such as the widespread distribution of ITNs and IRS, as well as increased community awareness and seasonal variations in transmission [18, 30, 31]. However, the persistence of asymptomatic malaria, even at lower levels, underscores the need for ongoing surveillance and targeted interventions to sustain these gains and further reduce transmission [18].

The study noted a gender disparity, with females having significantly lower odds of asymptomatic malaria compared to males (AOR: 0.20, 95% CI: 0.05–0.68; p = 0.010). This suggests that further gender-specific risk factors may play a role in the gendered distribution of asymptomatic malaria. Our results are consistent with a study conducted in Cameroon [32]. Similarly, previous research has shown that women are more vulnerable to malaria due to biological factors like pregnancy and HIV prevalence, as well as social roles that increase exposure [33]. In contrast, other studies have reported a higher prevalence of asymptomatic malaria among males. For instance, studies conducted in India, Tanzania, and Ghana found a higher prevalence of asymptomatic parasitemia in males [7, 34, 35]. These patterns have been attributed to factors such as increased outdoor exposure and gender-specific behaviors that increase the risk of mosquito contact [35, 36]. These variations highlight the importance of considering local environmental, socioeconomic and behavioral factors when examining gender-specific malaria risks. They also underscore the need for further research to understand the complex interplay of biological and environmental factors contributing to gender disparities in asymptomatic malaria prevalence.

Asymptomatic malaria prevalence varied across areas within the district, participants from Matoya were significantly more likely to have asymptomatic malaria compared to those from other areas, with an adjusted odds ratio (AOR) of 4.56 (95% CI 1.10–18.80; *p* = 0.036). Similarly, participants from Sikute had increased odds of asymptomatic malaria, with an AOR of 4.72 (95% CI 1.03–21.5; *p* = 0.045). These findings align with previous studies that have demonstrated the heterogeneous nature of malaria transmission dynamics across and within regions of SSA, highlighting varying rates of asymptomatic malaria among deferent vulnerable and demographic groups [7, 8, 25, 37].

No statistically significant associations were found between asymptomatic malaria and factors such as age, occupation, use of ITNs, IRS, socioeconomic status, or malaria prevention knowledge. These findings suggest that these factors may not independently influence asymptomatic malaria risk in the study population, or that their effects could be confounded by other unmeasured variables. Additionally, the lack of significance may be due to limitations such as sample size or variability in participants’ exposure and behavior, warranting further investigation in larger and more diverse populations.

This study had several limitations. The sample size of 370 participants was calculated based on an expected malaria prevalence of 50%. However, the actual observed prevalence in the study was 4.1%. The lower than the expected prevalence may have reduced the statistical power to detect associations between asymptomatic malaria infection and potential risk factors. Despite this, the sample size remained adequate to describe the presence of asymptomatic malaria in the population and contributes valuable baseline data for future research and surveillance. The reliance on RDTs and Giemsa-stained blood smears for malaria diagnosis may have limited the detection of all cases, particularly those with low parasite densities [38]. The observed discrepancy in prevalence between RDTs and microscopy, where RDTs yielded slightly higher rates, could be attributed to false-positive results, as RDTs may detect residual antigens even after parasite clearance. It is important to acknowledge the potential impact of histidine-rich protein 2 (HRP2) gene deletions on malaria diagnosis using RDTs. This study did not assess HRP2 gene deletions, which can lead to false-negative results with HRP2-based RDTs [39].

Future surveillance should consider monitoring HRP2/HRP3 deletions to ensure accurate malaria diagnosis and guide appropriate diagnostic tool use in endemic regions. PCR testing, known for its higher sensitivity, would have been a more accurate diagnostic method for detecting low-level parasitemia. However, due to resource constraints, PCR testing was not utilized in this study. Furthermore, the reliance on self-reported data for variables such as ITN usage and malaria prevention knowledge may introduce recall bias. Participants might overreport the use of preventive measures due to social desirability bias, potentially leading to an underestimation of asymptomatic malaria prevalence. Additionally, the cross-sectional design of the study limits the ability to establish causality between risk factors and asymptomatic malaria prevalence. Finally, A key limitation of this study was the low detection rate of gametocytes by microscopy, with only one participant presenting with detectable gametocytes. This limitation hindered the calculation of a meaningful geometric mean gametocyte density and may have led to an underestimation of the true burden of gametocyte carriage.

Despite these limitations, the implications of this study are significant for malaria control programs in Zambia and similar endemic regions. The study highlights a concerning prevalence of asymptomatic malaria in Matoya, Mwandi District, emphasizing the urgent need for enhanced surveillance and targeted interventions, particularly in high-prevalence areas like Matoya and among males. Asymptomatic malaria carriers contribute to ongoing transmission cycles, complicating efforts to monitor disease trends and evaluate control measures effectively. The failure to identify asymptomatic individuals is partly due to the limited availability of cost-effective diagnostic tools that are sufficiently sensitive to detect low levels of parasitemia such as PCR tests. This gap in diagnostic capability hinders the accurate detection of asymptomatic carriers, who often harbour low-density infections that are difficult to detect with standard methods [18, 40].

Future studies should employ more sensitive diagnostic tools, such as PCR, to improve detection of asymptomatic malaria. Longitudinal designs will be essential for understanding transmission dynamics and their contribution to the malaria burden. Investigating community-level factors, such as socioeconomic status, education, and healthcare access, can inform more effective intervention strategies. To reduce recall and social desirability bias, future research should incorporate objective measures like unannounced home visits to verify ITN use. Additionally, exploring sex-related immunity patterns across different transmission settings may guide targeted interventions for vulnerable demographic groups in endemic areas.

## Conclusion

This study highlights a low but notable prevalence of asymptomatic malaria, underscoring the ongoing risk of silent transmission in endemic settings. While RDTs and microscopy remain practical and widely used diagnostic tools, their limitations in detecting low-density infections suggest the need for integrating more sensitive methods such as PCR test in surveillance efforts, particularly in research and key surveillance sites. To strengthen malaria surveillance in Zambia, a combination of passive and active case detection, augmented by community-based screening and the use of digital health reporting platforms, could enhance real-time monitoring and response. Incorporating geospatial mapping tools and serological surveys may also help identify transmission hotspots and guide targeted interventions. Strengthened surveillance, particularly for asymptomatic cases, is essential to accelerate progress toward malaria elimination goals in Zambia and similar settings.

## Data Availability

The datasets used and/or analysed during the current study are available from the corresponding author on reasonable request.
